# The Next Frontier of Prematurity: Predicting Respiratory Morbidity During the First Two Years of Life in Extremely Premature Babies

**DOI:** 10.7759/cureus.23505

**Published:** 2022-03-26

**Authors:** Jered Weinstock, Xilie Xuchen, Maria Arroyo, Hector Aguilar, Ryan Kahanowitch, Maria J Gutierrez, Gustavo Nino

**Affiliations:** 1 Pediatric Pulmonology, Children’s National Hospital, Washington, DC, USA; 2 Pediatric Allergy, Immunology and Rheumatology, Johns Hopkins University School of Medicine, Baltimore, USA; 3 Pulmonary Medicine and Sleep Medicine, Children’s National Hospital, Washington, DC, USA

**Keywords:** pediatrics and neonatology, respiratory disease, extreme preterm care, bronchopulmonary dysplasia, prematurity

## Abstract

Background

Advances in perinatal and neonatal medicine have led to an increasing number of infants surviving extreme prematurity (≤27 weeks gestational age, GA). The goal of this study was to examine the respiratory outcomes after neonatal intensive care unit (NICU) discharge of this vulnerable population. We hypothesized that the rates of respiratory hospitalizations are disproportionally higher in the subset of infants born ≤27 weeks GA relative to premature infants born 28-32 weeks GA.

Methodology

A retrospective longitudinal study of severe premature children (≤32 weeks GA, n = 183) was conducted. We subdivided our sample into extremely preterm infants (≤27 weeks GA; n = 101) and those born very preterm (28-32 weeks GA; n = 82). Our main outcome was the presence of respiratory hospitalizations within 24 months of NICU discharge.

Results

Extremely premature infants had more than three times higher odds of respiratory hospitalization at 24 months relative to infants born 28-32 weeks GA (adjusted odds ratio = 3.4; 95% confidence interval = 1.8, 6.4; p < 0.01). The increased risk of respiratory hospitalization in extremely premature infants was independent of GA. Regression models identified that the duration of supplemental oxygen and Black/African American ethnicity were significant predictors of respiratory hospitalizations in both prematurity groups independent of gender and birth weight.

Conclusions

The results support that babies born ≤27 weeks GA represent a distinct high-risk group of severely premature infants that needs novel preventive strategies and targeted interventions to improve their respiratory outcomes after NICU discharge.

## Introduction

Advances in perinatal and neonatal medicine after the “post-surfactant era” have resulted in a dramatic improvement of survival rates for extremely premature infants over the past 30 years [1-3]. Surfactant is produced during the saccular phase of lung development [≈28-36 weeks gestational age, GA), following the initial alveolar-capillary formation in the canalicular phase (≈16-27 weeks GA) [1-3]. It is remarkable that after the development of surfactant therapy, most premature babies born during their saccular phase (>28 weeks of GA) can survive. Furthermore, although uncommon in some parts of the world, babies born lacking the entire third trimester of intrauterine life (born ≤27 weeks GA) nowadays have a good chance of surviving the newborn period and eventually being discharged home from the neonatal intensive care unit (NICU) [4-7]. The increasing availability of high complexity-level NICU care globally promises even further improvements in survival rates for the most immature and smallest infants with extremely low birth weights (BWs).

The clinical data regarding long-term impairments among extremely premature babies born ≤27 weeks GA after NICU discharge are scarce but concerning [7,8]. The rates of neurodevelopmental injuries, including severe intracranial hemorrhage, are significantly higher among very low BW infants [4,9-11]. Regarding the respiratory system, several factors put extreme survivors of prematurity at a much higher risk of severe complications. Babies born ≤27 weeks GA are not simply “smaller,” they are at the end of the canalicular stage of lung development. At this stage, the pulmonary gas exchanging units are highly vulnerable because they are still forming and vascularizing [1-3]. Therefore, advanced respiratory support is commonly needed for babies born ≤27 weeks GA, often resulting in longer exposure to iatrogenic therapies such as oxygen and mechanical ventilation in the NICU. The degree to which improvements in respiratory outcomes after NICU discharge can be achieved for most premature infants is unclear, especially in the post-surfactant era. Our team and others [8,12-17] have shown that despite surviving early respiratory complications, infants born severely premature (≤32 weeks GA) have very high rates of respiratory hospitalizations after NICU discharge. It is possible that extremely premature babies born at the limit of the viability of lung development have an even greater risk of respiratory morbidity. The most immature and smallest infants may represent a distinct high-risk category that needs to be recognized among infants born severely premature.

The goal of this study was to define the rates of respiratory hospitalization following NICU discharge among extreme survivors of prematurity born ≤27 weeks GA. We tested the hypothesis that the rates of acute respiratory illnesses leading to hospitalizations are disproportionally higher in the subset of infants born ≤27 weeks GA relative to premature infants born 28 to ≤32 weeks GA. We also examined early predictors of respiratory hospitalization in these two groups of premature infants. The results support that babies born ≤27 weeks GA are a distinct high-risk group of severely premature infants that needs novel preventive strategies and targeted interventions to improve their respiratory outcomes after NICU discharge.

## Materials and methods

Study design

**Figure 1:**
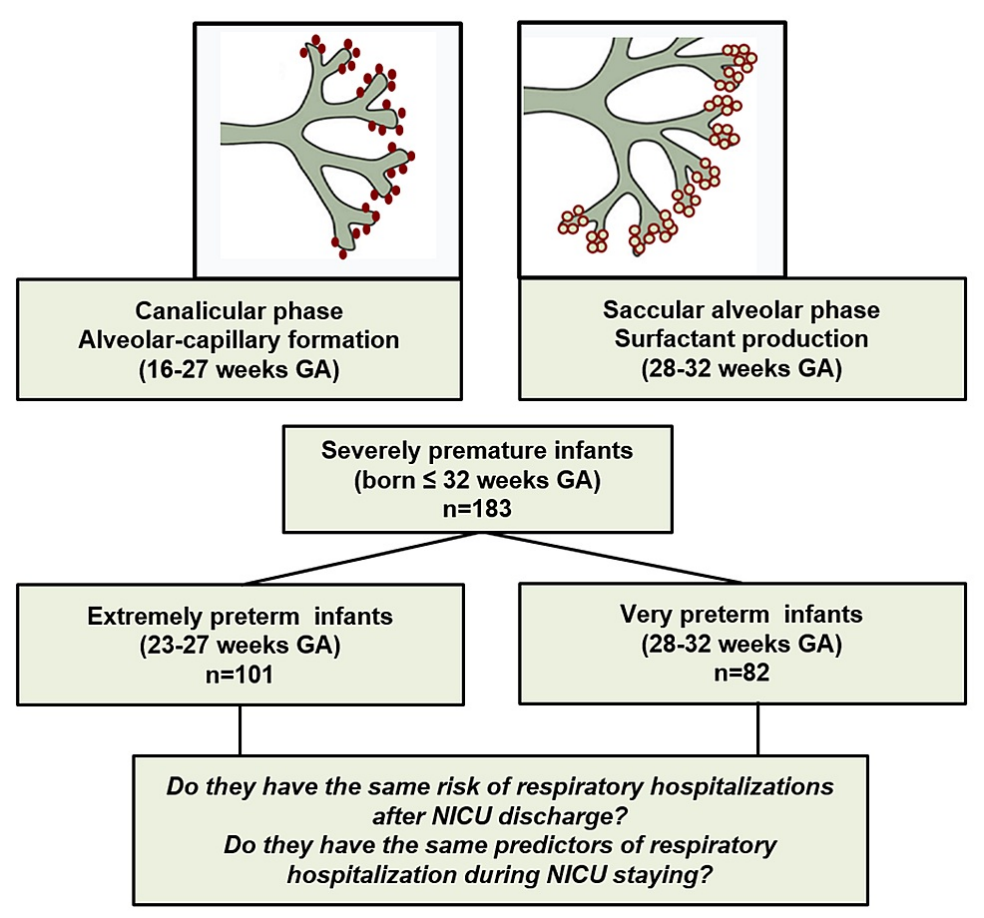
Study design and rationale. Respiratory outcomes during the first two years of life were contrasted in severely premature babies (n = 183) born extremely preterm at the end of the canalicular phase (≤27 weeks GA,  n = 101) and in very preterm babies born 28-32 weeks GA (n = 82). Predictors of respiratory outcomes in these subgroups were examined using regression models. GA: gestational age; NICU: neonatal intensive care unit

We conducted an analytical, single-center, longitudinal retrospective study that included premature infants ≤32 weeks GA admitted to the NICU at Children’s National Health System (CNHS) in Washington, DC. To test our main hypothesis, we separated individuals according to GA into the following two groups: extremely preterm babies born ≤27 weeks GA and those born very preterm between 28 and 32 weeks GA (Figure 1). We only included premature infants who had continued clinical care in CNHS for at least 24 months after discharge and had complete electronic medical record (EMR) data (NICU and outpatient) to ascertain outcomes and predictors of interest. We excluded premature newborns with (1) incomplete EMR data; (2) congenital conditions that may affect airway anatomy or lung development (such as congenital cyanotic heart disease, congenital diaphragmatic hernia, cystic fibrosis, airway abnormalities); (3) tracheostomy dependence; (4) immunodeficiency; and (5) neuromuscular disorders. The Institutional Review Board (IRB) of CNHS approved the study and granted a waiver of informed consent given that this research involved materials (data, documents, records, or specimens) collected solely for non-research purposes (clinical indications).

Outcome definitions

The main outcome was the binary presence (0 or ≥1 episode) of a respiratory illness leading to hospitalization following 12 months and 24 months of NICU discharge. We only counted as respiratory hospitalization those in which the primary complaint was any type of respiratory sign or symptom (e.g., cough, nasal/chest congestion, wheezing, respiratory distress, hypoxemia, etc.). We also recorded the binary need for admission to the pediatric intensive care unit (PICU) during acute respiratory illnesses. Bronchopulmonary dysplasia (BPD) was defined according to the National Heart, Lung, and Blood Institute workshop [3-5]. “No BPD” was defined as <28 days of supplemental oxygen. Mild BPD included infants who received oxygen or respiratory support for >28 days but were on room air at 36 weeks post-maturational age (PMA). Infants with moderate BPD required supplemental oxygen, <30% FiO_2_, at 36 weeks PMA. Severe BPD was classified as the use of >30% FiO_2_ and/or the need for mechanical support and/or positive pressure at 36 weeks PMA. Premature infants receiving nasal airflow ≥3 L per minute at 36 weeks PMA were also included in the severe BPD category.

Statistical analysis

Differences between groups on continuous variables were analyzed using the unpaired t-test or the Mann-Whitney U test, whichever was appropriate. Associations between categorical variables were analyzed using the chi-square test. To identify predictive factors of respiratory hospitalization, we built univariate and multivariate logistic regression models. Regression results are reported as coefficients (β), odds ratios (ORs), 95% confidence intervals (95% CIs), and respective p-values. All statistical tests were two-tailed, and the significance level used was p < 0.05. The data were analyzed using the Minitab Statistical Package version 19.1. (Minitab, Inc., State College, PA).

## Results

We included a total of 183 premature children born ≤32 weeks GA in this study. Our sample was divided into extremely preterm infants born ≤27 weeks GA (n = 101) and those born very preterm 28-32 weeks GA (n = 82). A comparison of all the demographic and clinical characteristics measured in these two groups is presented in Table 1. BW, Apgar scores, duration of respiratory support (oxygen mechanical and mechanical ventilation), chorioamnionitis, and BPD severity were significantly different in the two groups (Table 1). The proportion of Black/African American infants was greater in the extremely preterm group, and gestational diabetes was more commonly reported in those born 28-32 weeks GA (Table 1).

**Table 1:** Characteristics of the study subjects according to prematurity status. GA: gestational age; NICU: neonatal intensive care unit; BPD: bronchopulmonary dysplasia; PICU: pediatric intensive care unit; SD: standard deviation

Clinical variables	Born at ≤27 weeks GA (n = 101)	Born at 28-32 weeks GA (n = 82)	P-value
GA at birth (weeks, mean, SD)	24.6 (1.3)	30.0 (1.5)	<0.001
Male gender (%)	61.4	56.1	0.47
Black/African American (%)	68.3	52.4	0.03
Birth weight (g, mean, SD)	693 (165)	1357 (419)	<0.001
Cesarean section (%)	58.0	70.1	0.07
Apgar score at one minute (SD)	3.7 (2.1)	5.9 (2.2)	<0.001
Apgar score at five minutes (SD)	6.3 (2.1)	7.7 (1.5)	<0.001
Chorioamnionitis (%)	11.0	3.7	0.05
Gestational diabetes (%)	1.0	8.5	0.02
Maternal smoking (%)	7.9	2.4	0.09
Maternal use of recreational drugs (%)	7.9	3.7	0.21
Length of NICU stay (days, mean, SD)	114.7 (35.4)	58.5 (32.5)	<0.001
Respiratory rate at discharge (breaths per minute, mean, SD)	54.3 (10.6)	53.7 (12.3)	0.74
Proportion of subjects with BPD at discharge (%)	99.0	52.4	<0.001
Moderate/severe BPD at discharge (%)	80.2	30.5	<0.001
Proportion of subjects with oxygen at discharge (%)	48.5	12.2	<0.001
Duration of oxygen therapy (days, mean, SD)	105.7 (37.9)	36.1 (33.8)	<0.001
Duration of mechanical ventilation (days, mean, SD)	38.6 (26.3)	9.3 (15.7)	<0.001
≥1 week of mechanical ventilation (%)	91.1	34.1	<0.001
≥30 days of mechanical ventilation %	59.4	9.8	<0.001
Number of respiratory hospitalizations within 12 months (mean, SD)	1.1 (1.4)	0.5 (1.0)	0.003
Proportion of subjects with respiratory hospitalization within 12 months (%)	58.4	28.0	<0.001
Number of respiratory hospitalizations within 24 months (mean, SD)	1.6 (1.6)	0.7 (2)	<0.001
Proportion of subjects with respiratory hospitalizations within 24 months (%)	66.3	34.1	0.002
PICU admission within 24 months (%)	21.8	11	0.044

**Figure 2:**
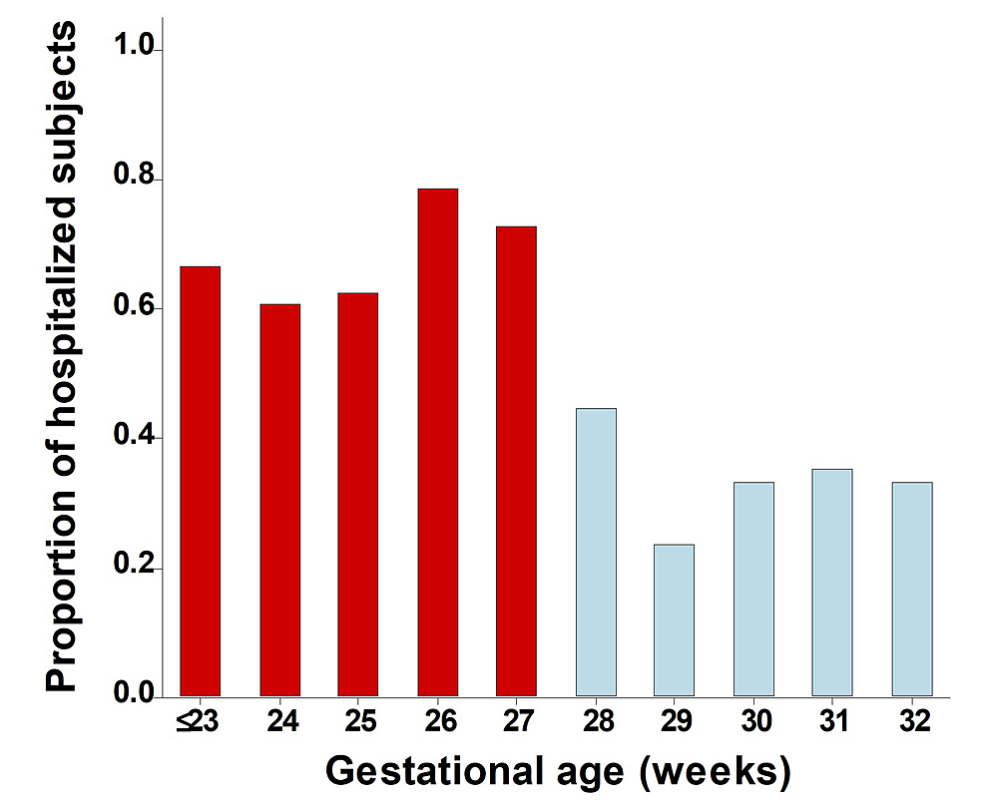
Respiratory outcomes according to gestational age. The proportion of respiratory hospitalizations during the first two years of life in extremely premature babies (≤27 weeks GA, red bars) and premature babies born 28-32 weeks GA (blue bars). GA: gestational age

In addition, we found that the group of extremely premature infants born ≤27 weeks GA had a significantly higher probability of respiratory hospitalizations within 12 months of NICU discharge (≤27 weeks GA; 58.4% versus 28-32 weeks GA; 28%, p < 0.01) and overall during 24 months of discharge (≤27 weeks GA; 66.3% versus 28-32 weeks GA; 34.1%, p < 0.01). As shown in Figure 2, the link between GA and the probability of respiratory hospitalization after NICU discharge was largely defined by the cut-off of ≤27 weeks GA as the risk was similar within each group independently of specific GA (Figure 2). After adjusting by gender and race/ethnicity (Black/African American as reference), we found that the risk of respiratory hospitalization was more than three times higher in premature infants ≤27 weeks GA relative to the infants born 28-32 weeks GA, both at 12 months (adjusted OR = 3.3; 95% CI = 1.7, 6.3; p < 0.01) and at 24 months of NICU discharge (adjusted OR = 3.4; 95% CI = 1.8, 6.4; p < 0.01).

We next examined the early clinical predictors of respiratory hospitalizations after 24 months of NICU discharge in extremely premature infants born ≤27 weeks GA and in those born 28-32 weeks GA. In the group of extremely preterm infants, we found that the number of days on supplemental oxygen, the diagnosis of moderate or severe BPD, and Black/African American ethnicity were significant predictors of early-life respiratory morbidity (Table 2). In the group of very preterm infants born 28-32 weeks GA, the number of days on supplemental oxygen was a significant predictor of subsequent respiratory hospitalizations (Table 2). Multivariate analyses showed that the number of days on supplemental oxygen and Black/African American ethnicity was significantly associated with respiratory hospitalizations in infants born 28-32 weeks GA and in extremely premature babies, independent of gender and BW (Table 3).

**Table 2:** Predictors of respiratory hospitalization within 24 months of NICU discharge in premature infants. *Positive values of β imply a positive association and negative values imply a protective association. Bold p-values are considered significant. GA: gestational age; NICU: neonatal intensive care unit; BPD: bronchopulmonary dysplasia

Predictors	≤27 weeks GA (n = 101)		28-32 weeks GA (n = 82)	
	Regression coefficient (β)*	P-value	Regression coefficient (β)*	P-value
Duration of oxygen therapy (total days)	0.014	0.025	0.017	0.018
Mechanical ventilation (total days)	0.009	0.280	0.015	0.289
Moderate/severe BPD at discharge	1.13	0.028	0.861	0.083
GA at birth (weeks)	0.124	0.458	-0.050	0.752
Birth weight (g)	-0.0008	0.540	-0.0002	0.749
Male gender	-0.024	0.956	0.287	0.545
Black/African American	1.24	0.006	0.736	0.125
Cesarean section	0.131	0.758	0.321	0.542
Apgar at one minute	0.015	0.881	0.005	0.962
Apgar at five minutes	0.066	0.522	-0.271	0.084
Chorioamnionitis	-0.961	0.138	-0.04	0.976
Maternal gestational diabetes	-13	0.967	0.405	0.613
Maternal smoking	1.35	0.217	-12	0.959
Maternal use of recreational drugs	1.35	0.217	-13	0.967

**Table 3:** Multivariate predictive model of respiratory hospitalization within 24 months of NICU discharge in premature infants. *Positive values of β imply a positive association and negative values imply a protective association. Bold p-values are considered significant.\ GA: gestational age; NICU: neonatal intensive care unit

Predictors	≤27 weeks GA (n = 101)		28-32 weeks GA (n = 82)	
	Regression coefficient (β)*	P-value	Regression coefficient (β)*	P-value
Constant	-3.20		-1.9	
Duration of oxygen therapy (total days)	0.01912	0.024	0.019	0.012
Birth weight (g)	0.00175	0.335	-0.00017	0.797
Black/African American	1.222	0.009	1.04	0.049
Male gender	-0.151	0.753	0.237	0.650

**Figure 3:**
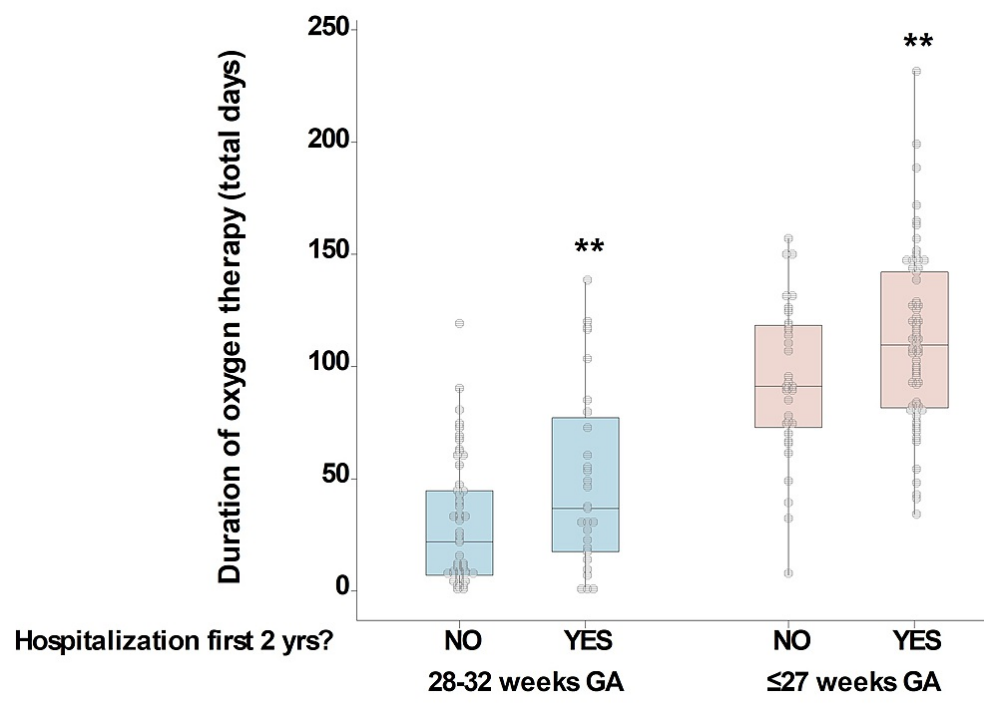
Oxygen therapy according to extreme prematurity and respiratory outcomes. Boxplots represent the days on supplemental oxygen in individuals with or without respiratory hospitalizations during the first two years separated as extremely premature infants (≤27 weeks GA, pink boxes) and premature infants born 28-32 weeks GA (blue boxes). **p < 0.01. GA: gestational age

To better understand the link between oxygen supplementation and the risk for respiratory hospitalization, we next compared the number of days on oxygen in our study groups. As shown in Figure 3, we found that the total number of days on supplemental oxygen was significantly greater in individuals with respiratory hospitalizations after NICU discharge in extremely premature infants (median of 110 days on oxygen in hospitalized versus 91 days in non-hospitalized; p < 0.01) and in those born 28-32 weeks GA (median of 36.5 days on oxygen in hospitalized versus 21.5 days in non-hospitalized; p < 0.01). Collectively, these results indicate that the number of days on oxygen is the strongest predictor of respiratory hospitalizations during early life in both very preterm and extremely preterm infants.

## Discussion

The goal of this study was to examine the respiratory outcomes after NICU discharge in infants surviving extreme prematurity (≤27 weeks GA). This is a high-impact research topic because the number of extreme prematurity survivors has increased globally and is projected to grow further in the future [4-7]. Our results demonstrate that extremely preterm infants have disproportionally high rates of respiratory hospitalizations. Specifically, infants born ≤27 weeks GA had more than three times higher odds of respiratory hospitalization at 24 months relative to very preterm infants born 28-32 weeks GA. Most infants born extremely premature had at least one respiratory hospitalization during the study period (66.3%), which was significantly greater than in those born 28-32 weeks GA (34.1%). The increased risk of respiratory hospitalization in extremely preterm infants was independent of GA. This indicates that 27-28 weeks GA is an important milestone in intrauterine lung development that determines individual risk for future respiratory morbidity, even after NICU discharge. Collectively, our results indicate that premature children born ≤27 weeks GA are a distinct high-risk group, and thus there is a critical need to better understand the pathological mechanisms of extreme prematurity to develop specific preventive strategies and interventions for this vulnerable population.

There is ample clinical data to demonstrate that premature infants have worse respiratory outcomes than full-term infants, especially regarding increased hospitalization rates early on in life in infants that have also developed BPD [18-21]. In addition to BPD, studies suggest that there may be other mechanisms at play regarding the pulmonary outcomes in extremely premature infants, including immature autonomic control [16], immune development [22], ventilatory responses, and cardiorespiratory coupling [23,24]. In addition, due to arrest in a critical early stage of lung development (canalicular phase), the lungs of infants born ≤27 weeks GA are much less competent for gas exchange [1,3,25], which extends oxygen supplementation requirement and potential iatrogenic damage. In support of this, in this study, we found that oxygen supplementation was disproportionally greater among extremely preterm infants, particularly in those with subsequent respiratory hospitalizations. This agrees with prior studies reporting that oxygen supplementation >120 days is an independent predictor of prolonged hospitalizations or readmissions for lower respiratory tract infections in premature infants [17,26]. We believe that premature infants born ≤27 weeks GA have a much higher risk of long-term complications and respiratory hospitalizations during the first two years of life due to additional pathogenic mechanisms, including abnormal development of the lungs and additional components of the respiratory system (e.g., autonomic and ventilatory control), as well as iatrogenic factors during prolonged NICU hospitalizations.

It is relevant to consider that what happens during lung development in extremely premature infants during their NICU stay has important implications on their later overall health and prognosis. Therefore, it is important to stratify these patients into risk categories to be able to predict long-term outcomes. As our study shows, babies born ≤27 weeks GA should be placed in a different severity category because they have a much greater risk of respiratory hospitalizations and may require new strategies to improve their outcomes. Long-term effects of extreme prematurity may also be reflected in deficits in lung function, [27-29], as well as pulmonary vascular disease, often associated with severe pulmonary hypertension [30]. Overall, given the exceedingly high risk for severe complications in extremely premature infants, it is critically important to have a detailed assessment of possible complications in this high-risk group, as well as intense surveillance by a specialized multidisciplinary team during and after NICU discharge.

Our study has several strengths and some limitations. We included a relatively large cohort of children born extremely premature. We were able to investigate longitudinal outcomes during the 24 months after discharge given that our institution encompasses the largest centralized system in Washington, DC, which connects outpatient centers and emergency rooms in inner-city and suburban areas. The main limitation of this study is the retrospective collection of clinical data. In addition, it is important to emphasize that the study was conducted in a specialized, tertiary referral hospital, which makes it likely that the patients included represent the extreme of the spectrum of severity of infants born premature, which could limit the generalization of results to other contexts. Finally, because EMR information did not provide a complete medical history in all cases, we did not include in the multivariate analyses some potentially important predictors of recurrent respiratory illnesses, such as eczema, socioeconomic status, and environmental factors (e.g., smoking and daycare attendance). Finally, as is the case for other observational epidemiologic studies, residual confounding cannot be excluded, so interpretation of our results needs to be cautious.

## Conclusions

Our study identified that respiratory hospitalization rates are disproportionately higher in the subset of infants born ≤27 weeks GA compared to premature infants born 28-32 weeks GA. In fact, >60% of extremely premature infants had at least one respiratory hospitalization during the first two years of life. The population of survivors of extreme prematurity has increased dramatically in recent decades, and therefore, there is now a critical need to develop novel preventive strategies and specific interventions for the smallest and most immature infants. Like the pioneering discovery of surfactant therapy to allow survival of babies born during the saccular phase of lung development (28-32 weeks GA), we now need new therapies to improve outcomes in babies born before the end of the canalicular phase (≈27 weeks GA). This milestone would have a dramatic impact on public health by preventing respiratory hospitalizations after discharge from the NICU in this increasingly prevalent and highly vulnerable population.
